# Dipeptidyl Peptidase (DPP)-4 Inhibitor Impairs the Outcomes of Patients with Type 2 Diabetes Mellitus After Curative Resection for Colorectal Cancer

**DOI:** 10.1158/2767-9764.CRC-21-0042

**Published:** 2021-11-22

**Authors:** Akira Saito, Joji Kitayama, Hisanaga Horie, Koji Koinuma, Rie Kawashima, Hideyuki Ohzawa, Hironori Yamaguchi, Hiroshi Kawahira, Toshiki Mimura, Alan Kawarai Lefor, Naohiro Sata

**Affiliations:** 1 Department of Gastrointestinal Surgery, Jichi Medical University, Shimotsuke, Japan.; 2 Department of Oral and Maxillofacial Surgery, Jichi Medical University, Shimotsuke, Japan.; 3 Departments of Clinical Oncology and Gastrointestinal Surgery, Jichi Medical University, Shimotsuke, Japan.

## Abstract

Dipeptidyl peptidase IV inhibitor (DPP-4i) has been shown to act either as a promoter or as a suppressor for cancer. Although epidemiologic studies suggest that DPP-4i does not correlate with the development of malignancies, its effects on cancer metastases are controversial. We evaluated the impact of DPP-4i on postoperative outcomes of the diabetic patients with colorectal cancer and microscopic features of the resected tumors. In 260 consecutive patients with type 2 diabetes mellitus (T2DM) who underwent curative resection of colorectal cancer, the correlation between DPP-4i use and prognosis was retrospectively examined. Expression of Zeb1 on tumor cells and density of infiltrating immune cells were quantitatively evaluated with multicolor IHC in 40 tumors from DPP-4i users, 40 tumors from propensity score–matched users, and 40 tumors from nonusers. Postoperative disease-free survival (DFS) was significantly lower in 135 patients treated with DPP-4i compared with 125 nontreated patients [5-year DFS, 73.7% vs. 87.4%; HR, 1.98; 95% confidence interval (CI), 1.05–3.71; *P* = 0.035]. IHC revealed that the number of Zeb1^+^ tumor cells increased in tumors from DPP-4i–treated patients than tumors from nonusers (*P* < 0.01). The densities of CD3^+^ and CD8^+^ T cells were significantly lower in tumors from DPP-4i users (*P* < 0.01) with decreased density of tertiary lymphoid structures (*P* < 0.001). However, the density of M2-type tumor-associated macrophages with CD68^+^ CD163^+^ phenotypes was significantly higher (*P* < 0.01) in tumors from DPP-4i users. Exposure of colorectal cancer to DPP-4i may accelerate epithelial-to-mesenchymal transition (EMT) creating a tumor-permissive immune microenvironment, which might impair the outcomes of the patients with colorectal cancer and T2DM.

**Significance:**

DPP-4i has been shown to enhance the antitumor effects of immunotherapy. However, we found that DPP-4i significantly impairs the outcomes of patients with colorectal cancer who underwent curative resection, possibly through acceleration of EMT and creation of a tumor-permissive immune microenvironment. This suggests that DPP-4i must be used with caution until its safety is fully confirmed by further studies of the mechanistic effects on existing cancers in humans.

## Introduction

Colorectal cancer has become the third most common cancer worldwide and is the second largest cause of cancer-related death, despite a decreased incidence due to the introduction of colorectal cancer screening (1). Type 2 diabetes mellitus (T2DM) is well-known to increase the risk of development and worsen the outcomes of various cancers (2, 3), including colorectal cancer (4–6). However, recent clinical studies have suggested that the prognosis of the patients with cancer is notably dependent on the antidiabetic drugs used (7). In particular, a number of studies have suggested positive effects of metformin on the outcomes of the patients with cancer (8–10).

Dipeptidyl peptidase IV inhibitors (DPP-4i) prevent the degradation of glucose-dependent insulinotropic polypeptide (GIP) as well as glucagon-like peptide 1 (GLP-1), and are now widely used for the treatment of the T2DM due to the low risk of hypoglycemia (11, 12). However, DPP-4 cleaves not only incretin hormones but also many substrates including growth factors, chemokines/chemokines, neuropeptides, and vasoactive peptides (13, 14). Besides, DPP-4 is a T-cell differentiation antigen (CD26) and plays a pivotal role in the immune response (15). These facts suggest that the long-term use of DPP-4i may modulate cancer cell properties and tumor immunity, which may affect the outcomes of the patients with cancer who also have T2DM.

Early clinical studies suggested an increased risk of malignancies in patients taking DPP-4i, especially pancreatic and thyroid cancers (16, 17). However, based on the results of recent epidemiologic studies, it is generally considered that the incidence of cancers is not significantly related to the use of DPP-4i by patients with diabetes (18–20). Meta-analyses including large population–based cohort studies have shown that DPP-4i does not significantly affect the outcome of patients with colorectal cancer and there is a trend toward reduction in the development of colorectal cancer (19, 21, 22). Consistently, preclinical studies have shown suppressive effects of DPP-4i on colorectal carcinogenesis (23–25). However, DPP-4i has been shown to either suppress (26, 27) or promote (28–31) tumor metastases and the exact effects of DPP-4i on already existing cancers are not clearly understood.

The aim of this study was to clarify the outcomes of patients with T2DM who underwent curative surgery for colorectal cancer and to assess the impact of treatment with DPP-4i on their postoperative clinical course. Multicolor IHC studies were performed using tissue sections from resected colorectal cancer to characterize the phenotypes of tumor cells and tumor-infiltrating immune cells in patients treated with or without DPP-4i.

## Materials and Methods

### Patients

From April 2010 to March 2020, 1,696 patients underwent curative colectomy with curative intent with stage I–III colorectal cancer in the Department of Surgery, Jichi Medical University Hospital (Shimotsuke, Japan). Of these, 260 patients (15.3%) were diagnosed with T2DM and 135 patients (51.9%) were treated with hypoglycemic medications, including DPP-4i, at the time of surgery. Data for gender, age, medical history, treatment of diabetes, surgical procedure, preoperative laboratory test results, pathologic evaluation (histologic type, depth of tumor, nodal metastasis, vascular invasion, lymphatic invasion), and postoperative outcomes were extracted for these patients from the electronic medical records after the patients provided written informed consent. This study was approved by the Institutional Review Board of Jichi University Hospital (Shimotsuke, Japan; approval no. clinic19–190) and was conducted in accordance with the Declaration of Helsinki guidelines.

### Antibodies and Reagents

mAbs to CD3 (MA5–14524, clone SP7, dilution 1:150) were purchased from Thermo Fisher Scientific and mAb to CD8α(66868–1-lg, clone 1G2B10, dilution 1:2,000)was obtained from ProteinTech Group. mAbs to CD68(ab955, clone KP1, dilution 1:100), CD163 (ab156769, clone OTI2G12, dilution 1:300), CD20 (ab9475, clone L26, dilution 1:100), Zeb1(ab181451, clone 2A8A6, dilution 1:200) CXCR4 (ab124824, clone UMB2, dilution 1:500), Ki67(ab16667, clone SP6, dilution 1:500), and pan Cytokeratin(ab7753, clone C-11, dilution 1:400) were from purchased Abcam. Signal enhancer HIKARI for Immunostain Solution B, antibody dilution buffer, and blocking solution One-Histo (06349–64) were purchased from Nacalai Tesque.

### Multiplex IHC

Among the 135 surgically resected colorectal cancer tumors from patients treated with DPP-4i, the latest 40 specimens were used for immunostaining. Samples from another 40 patients not receiving DPP-4i were selected from 125 cases by propensity score matching method and used as a control group in immunostaining. Age, tumor site, histologic type, pT category, pN category, p stage, adjuvant, and metformin intake, were selected for property score matching. All specimens were fixed in formalin, embedded in paraffin, cut into 4-μm–thick sections, and then used for IHC as well as hematoxylin and eosin (H&E) staining. The staining protocol has been described previously (32). In brief, after deparaffinization in xylene and rehydration in a graded series of ethanol immersion baths, sections were washed with distilled water for 5 minutes. Following deparaffinization, slides were stained with hematoxylin for 1 minute and then coverslips applied with VectaMount AQ Aqueous Mounting Medium (code H-5501, Vector Laboratories), followed by whole tissue scanning using OlyVIA SLIDEVIEW VS200 (Olympus). After imaging, coverslips were removed by immersion in Tris-buffered saline with Tween 20. Endogenous peroxidases were blocked using 0.3% hydrogen peroxide for 30 minutes. For antigen retrieval, the sections were processed by microwave method in 10 mmol/L sodium citric buffer (pH 6.0) for 10 minutes. After washing in TBST, a blocking agent (Blocking One Histo) was used to prevent nonspecific binding for 10 minutes. The sections were then incubated with primary antibodies for 30 minutes at room temperature. The sections were thoroughly washed with TBST and incubated using the Histofine Simple Stain PO(M) kit for either rabbit or mouse (Nichirei), and primary antibody binding was visualized using ImmPACT AMEC Red Substrate Kit (Vector Laboratories). The slides were again coverslipped in VectaMount AQ Aqueous Mounting Medium and scanned using OlyVIA SLIDEVIEW VS200.

After scanning, the coverslips were removed in TBST and treated for 3-Amino-9-ethylcarbazole (AEC) for destaining and antibody stripping. In summary, the stained slides were dehydrated in an alcohol gradient of 90% ethanol, 80% ethanol, and 70% ethanol for every 2 minutes. Slides were incubated until no visible AEC reaction product remained. After rehydration, antibodies were eluted by incubating sections in 10 mmol/L sodium citrate buffer (pH 6.0) for 10 minutes using the microwave method. The tissues were then restained, starting again with the blocking step, as described above. Complete stripping of antibodies and signals throughout all cycles was confirmed (Supplementary Fig. S1).

### Image Processing and Analysis

Image processing and analysis were performed as described previously (32). In brief, iteratively digitized images were coregistered so that cell features overlapped down to the single-pixel level, using a CellProfiler Version 2.1.1 pipeline, “Alignment_Batch.cppipe” available under GPLv2 (General Public License version 2.0). Visualization was performed via conversion of coregistered images to pseudo-colored single-marker images and merged by ImageJ Fiji (NIH, Bethesda, MD). In evaluation of the density of Zeb1^+^ cancer cells, the total number of positive cells was counted in randomly selected three square regions of interest (ROI) at the invasive front area (0.5 × 0.5 mm^2^) and the average was taken. In evaluation of the density of immune cells, three square ROIs (1.0 × 1.0 mm^2^) were randomly selected in the tumor stroma, and cell counting was performed with two different images of the same area, that is, CD3 ^+^ T cells or CD68^+^ macrophages were counted with single staining images, while CD3^+^CD8 ^+^ T cells or CD68^+^CD163^+^ macrophages were counted with double staining images. Tertiary lymphoid structures (TLS) were defined as a cluster of CD3^+^ T cells associated with CD20^+^ B cells, which often formed germinal centers (GC). The total number of TLS with or without a GC was counted in five randomly selected areas in stroma. Analysis was performed by two investigators who were blinded to clinical outcomes.

### Statistical Analysis

Statistical analyses were performed using Graph Pad Prism8. Statistical difference in clinical and pathologic factors was evaluated with the Mann–Whitney *U* test or Fisher exact test. Disease-free survival (DFS) was calculated using the Kaplan–Meier method and differences were evaluated using the log-rank test. In all analyses, the standard of significant difference was set as *P* < 0.05.

### Data Availability Statement

Data were generated by the authors and are available on request.

## Results

### DPP-4i is Associated with Poor Outcomes in Patients with colorectal cancer and T2DM

Table 1 shows the clinical and pathologic characteristics of 260 patients with T2DM treated with and without DPP-4i who underwent curative surgery for colorectal cancer. There were no significant differences in gender, tumor site, histologic type, HbA1c levels, pathologic stage between the two groups, although patients treated with DPP-4i tended to be older (*P* = 0.056). Ratios of patients who took metformin or received adjuvant therapy were similar in the two groups. Diabetic duration and the rate of complications did not differ (Supplementary Table S1).

**TABLE 1:** Patients with colorectal cancer treated with and without DPP-4i

Variables		DPP-4i(+) (*n* = 135)	DPP-4i(−) (*n* = 125)	*P*
Age		71 (44–91)	69 (42–88)	0.056
Gender	Male	87	87	0.38
	Female	48	38	
Tumor site	Right	47	41	0.73
	Left	88	84	
Histologic type	tub/pap	129	119	0.92
	por/muc	6	6	
pT Stage	t1	30	34	0.9
	t2	25	16	
	t3	50	42	
	t4	30	33	
pN Stage	n0	90	85	0.8
	n1	34	31	
	n2	10	8	
	n3	1	1	
p-Stage	Ⅰ	48	47	0.2
	Ⅱ	42	37	
	Ⅲ	45	41	
p-Lymphatic invasion	Yes	57	61	0.29
	No	77	63	
	Unknown	1	1	
p-Vascular invasion	Yes	39	40	0.58
	No	95	84	
	Unknown	1	1	
Adjuvant therapy	Yes	26	22	
	No	109	103	
Metformin intake	Yes	30	28	0.97
	No	105	97	
HbA1c		6.8 (5.7–9.0)	6.8 (5.5–9.9)	0.89

NOTE: *P* values were analyzed with Mann–Whitney *U* test or Fisher test.

**FIGURE 1:**
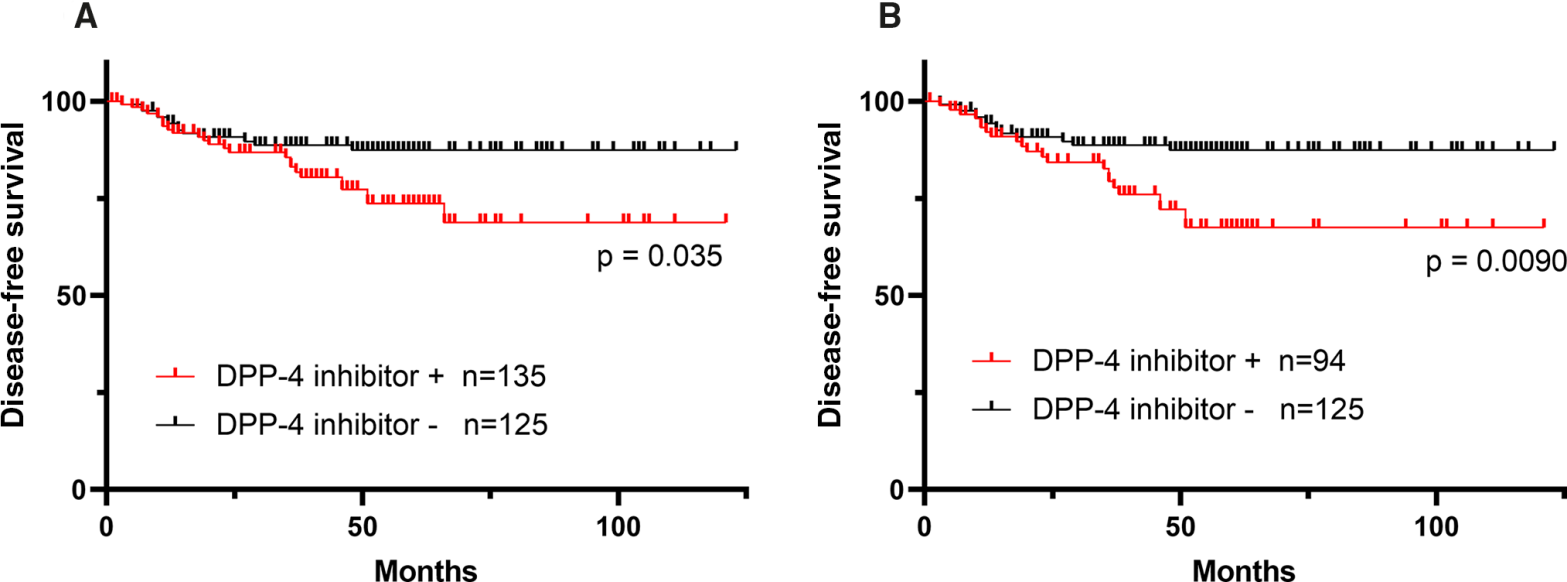
**A,** DFS after surgery in patients treated (*n* = 135) or not treated (*n* = 125) with DPP-4i was evaluated using the Kaplan–Meier method and *P* value was calculated using the log-rank test. **B,** DFS of 94 patients who were confirmed to have continued DPP-4i treatment after colectomy compared with DFS of patients not treated.

Among patients treated with DPP-4i at the time of surgery, recurrence occurred in 25 of 135 (19%) within median follow-up of 3.5 years, while 14 of 125 (11%) patients not treated with DPP-4i recurred over a similar follow-up period (median = 4.2 years). The DFS of patients treated with DPP-4i was significantly shorter than in untreated counterparts with HR = 1.98 and 95% confidence interval (CI), 1.05–3.71 (5 year DFS, 73.7% vs. 87.4%; *P* = 0.035; Fig. 1A). Multivariate analysis including pT and pN stages showed that DPP-4i intake was independently associated with a shorter DFS (*P* = 0.043; Table 2). In 94 of 135 patients, DPP-4i was confirmed to have been continued after surgery and their DFS was also shorter than those not treated with DPP-4i with a more pronounced difference [HR, 2.44; 95% CI, 1.22–4.62; *P* = 0.0090; Fig. 1B).

**TABLE 2:** Univariate and multivariate analyses of the correlation between variables and DFS of patients with colorectal cancer

	Univariate analysis		Multivariate analysis	
Variable	HR (95% CI)	*P*	HR (95% CI)	*P*
Age	0.731 (0.390–1.369)	0.329		
Gender (Male/Female)	0.950 (0.484–1.862)	0.882		
Pathologic T-stage	8.992 (4.769–16.960)	<0.001	4.459 (1.219–16.317)	0.024
Pathologic N-stage	4.904 (2.421–9.970)	<0.001	8.992 (2.449–74.525)	<0.001
Adjuvant therapy	1.757 (0.753–4.102)	0.119		
DPP-4i use	1.995 (1.064–3.740)	0.034	9.293 (1.077–80.205)	0.043

NOTE: *P* values were calculated with Cox regression analysis.

Because metformin is known to have anticancer properties, we examined the effect of metformin intake on the outcome. Metformin alone significantly improved the DFS in this series (Supplementary Fig. S2A). Moreover, if used together with DPP-4i, it cancelled the effects to reduce the outcome of the patients with colorectal cancer (Supplementary Fig. S2B).

### DPP-4i Increased the Number of Zeb1^+^ Tumor Cells in Resected Specimens

**FIGURE 2:**
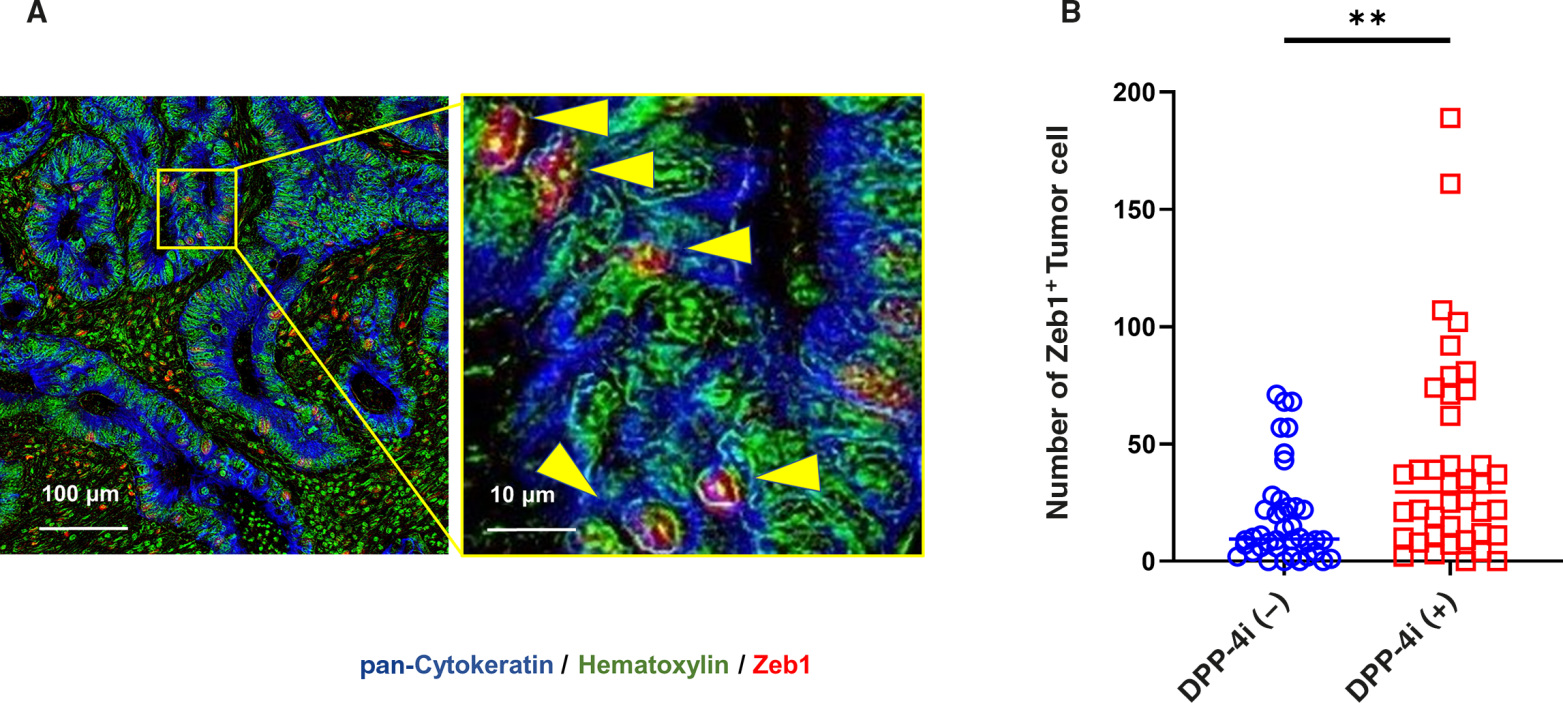
Representative images of multiplex immunostaining of Zeb1 and pan cytokeratin at the invasive front of colorectal cancer (CRC) from patients treated with DPP-4i (**A**). Images stained with hematoxylin (green), pan cytokeratin (blue), and Zeb1 (red) were merged. Arrow heads in **A** show Zeb1^+^ tumor cells. The average numbers of Zeb1^+^ tumor cells from patients treated with and without DPP-4i in square ROIs of 1.0×1.0 mm^2^ were compared (**B**). *P* values were calculated using Mann–Whitney *U* test. **, *P* < 0.01.

On the basis of the differences in patient outcomes, we examined whether the use of DPP-4i influences the cellular components in surgically resected tumor specimens using multicolor IHC. Table 3 shows the profiles of tumors from 40 patients treated with DPP-4i and from 40 patients not treated with DPP-4i selected by propensity score matching during the same period. DFS of the patients showed the same trend after propensity score matching (Supplementary Fig. S3). DFS of the patients treated with DPP-4i tended to be poor as compared with those without DPP-4i. First, we examined the number of tumor cells expressing Zeb1, a marker of the epithelial-to-mesenchymal transition (EMT), at the tumor-invasive front. Figure 2A shows a typical picture of multicolor staining. Pan-cytokeratin (blue) was expressed in most tumor cells forming tubular structures, while Zeb1 (red) was mainly detected in the stromal area. In some tumor cells in the parenchyma, however, Zeb1 was clearly colocalized with hematoxylin (green) positive nuclease with relatively reduced staining for pan-cytokeratin, which are considered to be tumor cells undergoing EMT. As shown in Fig. 2B and Supplementary Fig. S4, there were significantly more Zeb1^+^ tumor cells in tumors from patients treated with DPP-4i than from nontreated patients [median, 29.0 (0–189) vs. 9.0 (0–71); *P* < 0.01].

**TABLE 3:** Characteristics of colorectal cancer in patients with or without DPP-4i use evaluated by IHC

Variables	DPP-4i(+) (*n* = 40)	DPP-4i(−) (*n* = 40)	*P*
Tumor site			
Cecum/ascending/transverse	16	19	0.53
Descending/sigmoid	12	11	
Rectum	12	10	
Histologic type			
tub1/tub2/pap	39	39	>0.99
muc/por	1	1	
pT category			
t1	6	5	0.64
t2	7	7	
t3	22	21	
t4	5	7	
pN category			
n(−)	25	24	>0.99
n(+)	15	16	
p-stage			
Ⅰ	12	11	0.83
Ⅱ	13	13	
Ⅲ	15	16	
Metformin intake			
Yes	9	11	0.80
No	31	29	

NOTE: *P* values were analyzed with Mann–Whitney *U* test or Fisher tests.

### DPP-4i Reduced the Density of Tumor-Infiltrating Lymphocytes in Colorectal Cancer

**FIGURE 3:**
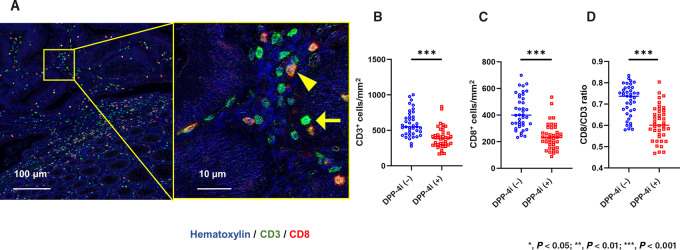
Representative images of multiplex immunostaining of TILs in colorectal cancer (CRC) treated with DPP-4i (**A**). Images stained for CD3 (green) and CD8 (red) were merged. Arrow and arrowhead show a representative T cells of CD3^+^CD8^−^ and CD3^+^CD8^+^ phenotypes, respectively. The average numbers of CD3^+^ TILs (**B**), CD3^+^CD8^+^ TILs (**C**) and the ratios of CD8^+^/CD3^+^ (**D**) in square ROIs of 0.5×0.5 mm^2^ were compared for colorectal cancer from patients treated with and without DPP-4i. *P* values were calculated using Mann–Whitney *U* test. ***, *P* < 0.001.

Because it is known that DPP-4i modulates various immunologic components, we next examined the density and phenotype of immune cells in resected tumor specimens. Tumor-infiltrating lymphocytes (TIL) were examined by immunostaining using mAbs to CD3 and CD8. CD3^+^ T cells were visualized as green dots with single stain imaging, while all CD8 ^+^ cells were colocalized with CD3^+^ cells and detected as yellow dots in dual stain imaging (Fig. 3A)

As shown in Fig. 3B and Supplementary Fig. S5A, the median number of CD3^+^ T cells infiltrating tumors in patients treated with DPP-4i was 388 (169–834)/mm^2^, which was significantly fewer than in tumors from patients not treated with DPP-4i (median, 550, 278–1,003/mm^2^, *P* < 0.01). However, the densities of CD8^+^ T cells were further reduced in tumors from patients treated with DPP-4i [median, 33.0 (88–535)/mm^2^ vs. 400 (232–699)/mm^2^; *P* < 0.01; Fig. 3C], and CD8^+^/CD3^+^ ratios were significantly lower in tumors from patients treated with DPP-4i [60.1% (46.8%–80.4%) vs. 73.7% (57.9%–83.4%); *P* < 0.001; Fig. 3D].

### DPP-4i Increased the Density of M2-type TAMs in Colorectal Cancer

**FIGURE 4:**
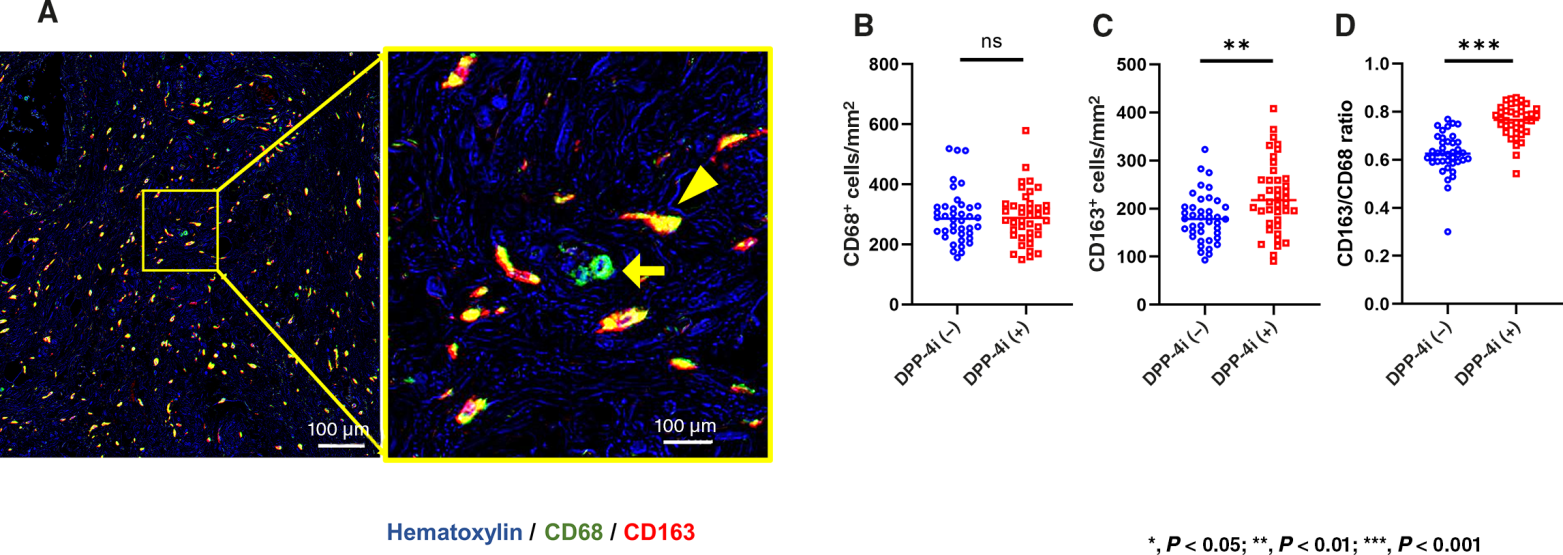
Representative images of immunostaining of TAMs in colorectal cancer (CRC) tumors from patients treated with DPP-4i (**A**). Images stained for CD68 (green) and CD163 (red) were merged. Arrow and arrowhead show representative macrophages with CD68^+^CD163^−^ and CD68^+^CD163^+^ phenotype, respectively. The average numbers of CD68^+^ TAMs (**B**), CD68^+^CD163^+^ TAMs (**C**), and the ratios of CD163^+^/CD68^+^ (**D**) in square ROIs of 0.5×0.5 mm^2^ were compared for colorectal cancers from patients treated with and without DPP-4i. *P* values were calculated using the Mann–Whitney *U* test. **, *P* < 0.01; ***, *P* < 0.001.

Figure 4A shows a representative dual color image of TAMs stained with mAbs to CD68 and CD163. The CD163^+^ cells were mostly merged with CD68^+^ cells, although not merged perfectly probably due to the different locations of antigens in TAMs. The total number of CD68^+^ TAMs did not differ between two groups [median, 288 (150–579)/mm^2^ vs. 285.8 (156–519)/mm^2^; *P* = 0.76; Fig. 4B; Supplementary Fig. S5B]. However, the density of CD68^+^ CD163^+^ macrophages in tumors from patients treated with DPP-4i was significantly higher than in their counterparts [217.7 (96.7–408)/mm^2^ vs. 178.5 (93–323)/mm^2^; *P* < 0.001; Fig. 4C]. Thus, the ratios of M2-type macrophages defined by CD163^+^ cells /CD68^+^ cells were significantly elevated in tumors from patients treated with DPP-4i [76.7% (54.2%–85.9%) vs. 62.5% (30.0%–76.9%); *P* < 0.001; Fig. 4D].

### DPP-4i Decreased the Density of TLS in Colorectal Cancer

**FIGURE 5:**
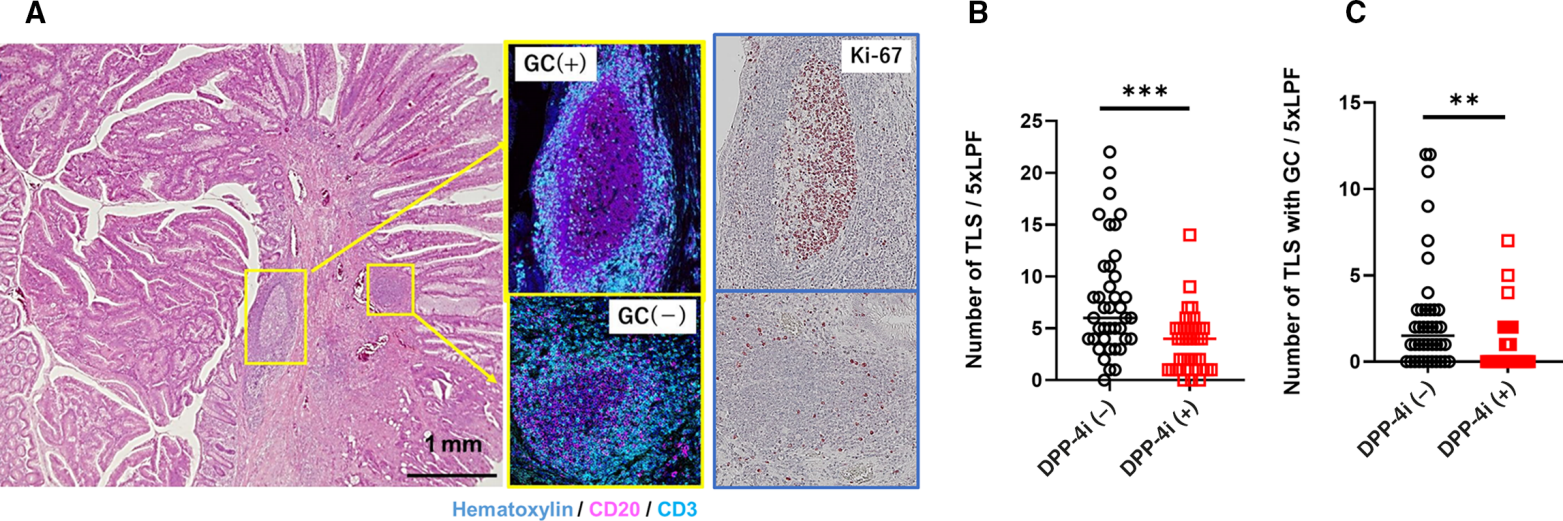
**A,** Representative image TLS with or without GC formation in colorectal cancer (CRC) from patients without DPP-4i treatment. H&E staining and immunostaining of CD20^+^ B cells (purple) and CD3^+^ T cells (blue). Immunostaining of Ki-67 of the TLS was performed in continuous tissue section (right figures). **B,** Total numbers of TLS (**B**) and TLS with GC formation (**C**) in five randomly selected low power fields (LPF; 40×) of colorectal cancer from patients treated with or without DPP-4i. *P* values were calculated using the Mann–Whitney *U* test. **, *P* < 0.01; ***, *P* <0.001.

TLS are ectopic lymph node–like structures that develop in the stroma of tumors due to an immune response against tumor antigens. As shown in Fig. 5A, many TLS were detected as aggregated CD20^+^ B cells surrounded by CD3^+^ T cells and some of the TLS formed clear GC in colorectal cancer. Ki-67 was highly stained in B cells in GC of TLS (Fig. 5A). The total numbers of TLS in five randomly selected fields were significantly fewer in tumors from patients treated with DPP-4i than in tumors from untreated patients (median 4, 0–14 vs. 6, 0–22; *P* < 0.001; Fig. 5B). Figure 5C shows the same trend in the number of TLS with GC (median 0, 0–7 vs. 2, 0–12; *P* < 0.001).

## Discussion

Recent meta-analyses and large-scale cohort studies have suggested that the use of DPP-4i does not significantly affect the outcome of the patients with cancer who also have T2DM (18–20, 22, 30, 33). Studies focused on colorectal cancer have shown that DPP-4i have no association with the incidence of colorectal cancer although its association does not reach statistical significance (19, 21, 22, 34, 35). Other retrospective studies with small sample size have suggested that DPP-4i intake is associated with even a favorable outcome in patients with colorectal cancer (36, 37). In this study, however, the use of DPP-4i in patients with colorectal cancer was clearly associated with a higher incidence of tumor recurrence and with a shorter DFS. The exact reason of this discrepancy is unclear yet, but might be dependent on the difference of patient selection. In past studies, many differences exist in patient backgrounds, such as the stage of colorectal cancer, observation period, and the timing of DPP-4i treatment. In our study, we focused on the patients who received curative resection and examined the effect of DPP-4i intake before surgery on postoperative outcome.

A recent observational study has shown that DPP-4i alogliptin increase the incidence of cancer in patients with T2DM and they described it might be explained by age (an elder in DPP-4i users; ref. 38). In our series, however, age was not significantly associated with DFS, although patients taking DPP-4i were marginally elder than DPP-4i nonusers. Therefore, it is unlikely that age is the major factor to affect the outcome of the patients with colorectal cancer. Instead, the difference in prognosis is considered to be dependent on the drug effect, because DPP-4i use is an independent prognostic factor with no differences in other background factors including stage, adjuvant treatment, and metformin intake.

The difference in results might be caused by the differences in the patient populations. In this study, we investigated postoperative outcomes after curative tumor resection and thus the shortened DFS in patients treated with DPP-4i is thought to be caused mainly by the subclinical microscopic spread of tumor at the time of surgery. IHC studies showed that the number of Zeb1^+^ tumor cells is significantly increased at the invasive front of tumors from patients treated with DPP-4i. Recent preclinical studies have shown that DPP-4i can increase viability and proliferation of tumor cells, and accelerates EMT through the CXCL12/CXCR4/mTOR axis both *in vitro* and *in vivo* (14, 30). We examined the expression of CXCR4 in these tissue samples and found that CXCR4^+^ tumor cells tended to be increased in DPP-4i–treated tumors (Supplementary Fig. S6). This is consistent with the results of this study and suggests the possibility that exposure of colorectal tumors to DPP-4i might enhance the development of micrometastases through the induction of EMT at the timing of surgery, resulting in poor overall outcomes.

More interestingly, the immune microenvironment in colorectal tumors was remarkably altered by DPP-4i. In tumors from patients treated with DPP-4i, the rate of M2-type macrophages, defined as CD163^+^ CD68^+^ cells, was significantly increased, although the density of the total CD68^+^ TAMs was not altered. Previous studies have shown that GLP-1 induces M2 polarization of human macrophages through the JNK–STAT3 pathway (39, 40). Taking these results into consideration, it is suggested that DPP-4i alters the balance of TAMs from favoring M1 to an M2 predominant state by increasing the concentration of GLP-1 in colorectal cancer tissue, which may have increased the rate of development of micrometastases in these patients. A recent study has consistently shown that the ratio of CD163^+^/CD68^+^ macrophages in tumor tissue is an independent prognostic factor for survival in patients with colorectal cancer (41).

In contrast, the number of effector TILs, defined as CD3^+^ CD8^+^ phenotype, was significantly reduced in tumors from patients treated with DPP-4i. Previous experimental studies have reported conflicting results about the effect of DPP-4i on lymphocyte trafficking to the tumor site. Barreira da Silva and colleagues have shown that DPP-4 inhibition preserved the active form of chemokine CXCL10, which enhanced T-cell accumulation and immunity, resulting in diminished growth of murine melanoma and colon cancer (27). Similar results have been reported for hepatocellular carcinoma and breast cancer in mice (42, 43). However, the function of DPP-4/CD26 in the human immune system was reported to be notably different from that in mice (44), and therefore results using murine models should be cautiously interpreted. Iwata and colleagues have reported that the addition of soluble CD26/DPP-4 enhances transendothelial migration of human T cells (45). Marx and colleagues have also reported that GLP-1 inhibits chemokine-induced migration of human T cells through the inhibition of PI3K activity (46). The results of this IHC study are consistent with their results and suggest that DPP-4 inhibition might exert opposite effects on T-cell accumulation in human tumors compared with rodents.

Another interesting finding is that the number of TLS is significantly reduced in patients with colorectal cancer treated with DPP-4i. TLS are ectopic lymphoid organs that develop in peripheral tissues and are considered to be privileged sites for the generation of effector T cells, memory B cells, and antibodies against tumor antigens (47, 48). The results in this study appear to be reasonable because early studies have shown that DPP-4/CD26 deliver potent costimulatory signals for T-cell activation (49) and that inhibition of DPP-4/CD26 suppresses T-cell proliferation and enhances production of immunosuppressive cytokines (50, 51). Given the fact that the number of TLS is associated with favorable outcomes of patients with colorectal cancer (52, 53), the impaired outcomes of patients treated with DPP-4i in this series may be partially dependent on the suppression of TLS formation through a direct effect on T lymphocytes.

The detailed mechanisms how DPP4i affects tumor–immune microenvironment remain unclarified. In the 80 tumors examined in this series, the number of Zeb1^+^ tumor cells showed weak negative correlations with the density of CD3^+^ CD8^+^ TILs while not correlated with density of TAMs (Supplementary Fig. S7A). The number of Zeb1^+^ tumor cells also showed weak negative correlation with the number of TLS. However, the number of TLS positively correlated with the density of CD3^+^ and CD8^+^ TILs (Supplementary Fig. S7B). These results suggest that the effects of DPP-4i to induce EMT in tumor cells might be partially related with the reduction of TLS in tumor tissues causing the reduced TILs. On the contrary, DPP-4i is supposed to directly affect the recruitment and differentiation of macrophages in tumor tissue which is independent on the effects on tumor cells.

In summary, the treatment of DPP-4i impairs the outcomes of patients with T2DM who underwent curative surgery for colorectal cancer. Histologic evaluation suggests that exposure to DPP-4i enhances the EMT of tumor cells and produces a tumor-permissive immune microenvironment in human colorectal cancer, although the mechanisms are not yet clearly defined. To the best of our knowledge, this is the first report to show an inverse association between treatment with DPP-4i and outcomes of patients with colorectal cancer. Data in previous studies suggest that DPP-4i potentially acts as either a tumor suppressor or promoter depending on the level of DPP-4/CD26 expression and its interaction in the tumor microenvironment (54). Although epidemiologic studies indicate no clear association between treatment with DPP-4i and the prognosis of patients with cancer and T2DM at this time, safety of this drug for patients with malignancies is an unsolved issue. Further investigations of the mechanistic link between DPP-4i and tumor biology are necessary, especially regarding any effects on the progression of tumor cells already present.

## Supplementary Material

Supplementary Data Supplementary Table 1 and Supplementary Figure1~7
