# Supplementary material for: Dipeptidyl Peptidase (DPP)-4 Inhibitor Impairs the Outcomes of Patients with Type 2 Diabetes Mellitus After Curative Resection for Colorectal Cancer
Source: Cancer Res Commun. 2021 Nov 22;1(2):106–14. doi: 10.1158/2767-9764.CRC-21-0042 (PMC9973397; doi:10.1158/2767-9764.CRC-21-0042)
Supplement: Supplementary Data — Supplementary Table 1 and Supplementary Figure1~7 [file crc-21-0042-s01.pdf]

Supplementary Table 1

Patients with colorectal cancer treated with and without DPP-4i

Variable	DPP-4i (+) (135)	DPP-4i (-) (125)	P-value
Diabetic duration (month)	120 (12-528)	120 (12-516)	.56
Diabetes-related complications			
Cardiovascular disease	13	18	.47
Cerebrovascular disease	16	15	.97
Retinopathy	12	11	.68
Nephropathy	13	9	.28
Neuropathy	1	1	>.99

P values were analyzed with Mann-Whitney' test or Fisher's tests.

Supplementary Figure 1

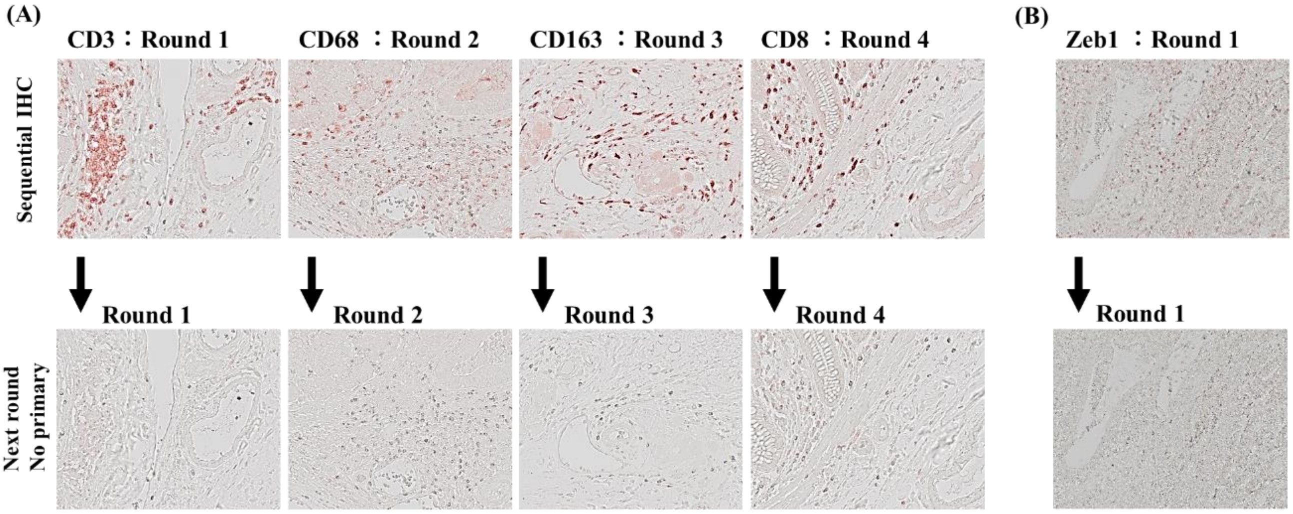

To validate antibody-stripping, staining was performed in the same number of rounds in corresponding shown in Table S1. Images represent chromogenic staining with labeled biomarkers and the number of round (top panel). Following AEC wash and antibody stripping, complete removal of antibody and signal was confirmed by incubating with only the detection reagent and AEC in the next sequential round (bottom panel)

Supplementary Figure 2

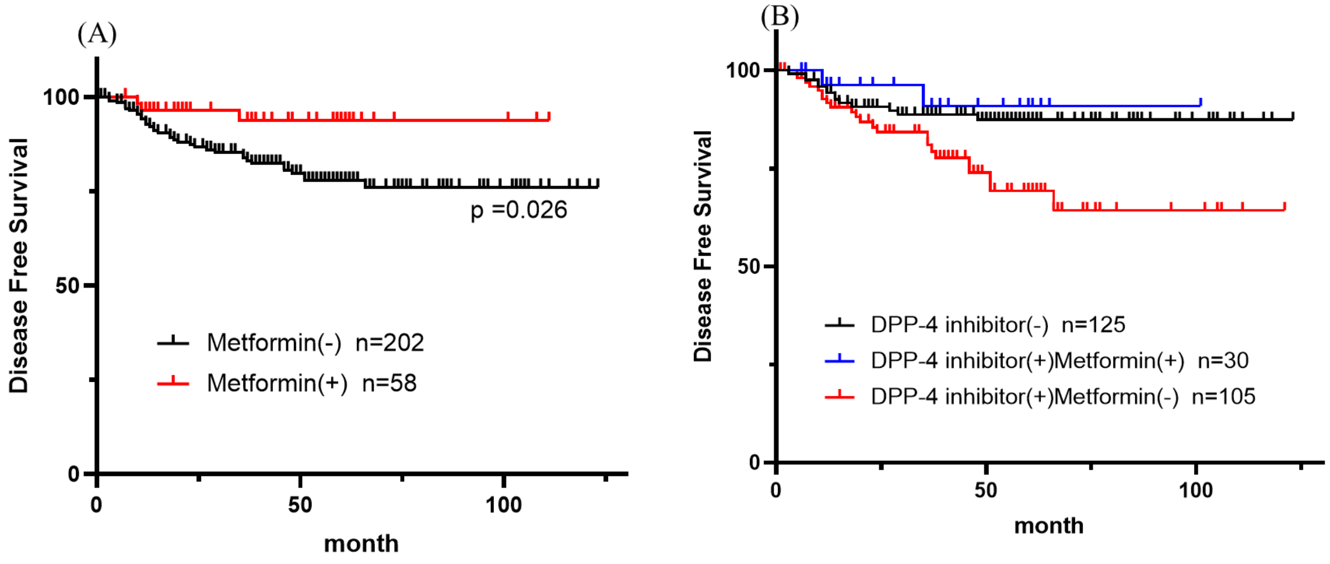

Metformin alone significantly improved the DFS in this series (A). When metformin was used together with DPP-4i, effects of DPP-4i on patient outcome were totally cancelled (B).

Supplementary Figure 3

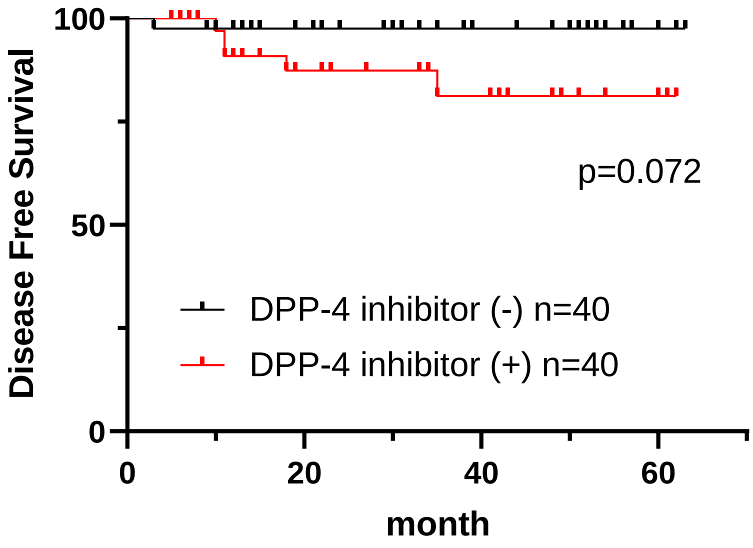

Outcome of the patients with CRC after propensity score matching. Disease free survival (DFS) of the patients treated with DPP-4i tended to be poor as compared with those without DPP-4i.

Supplementary Figure 4

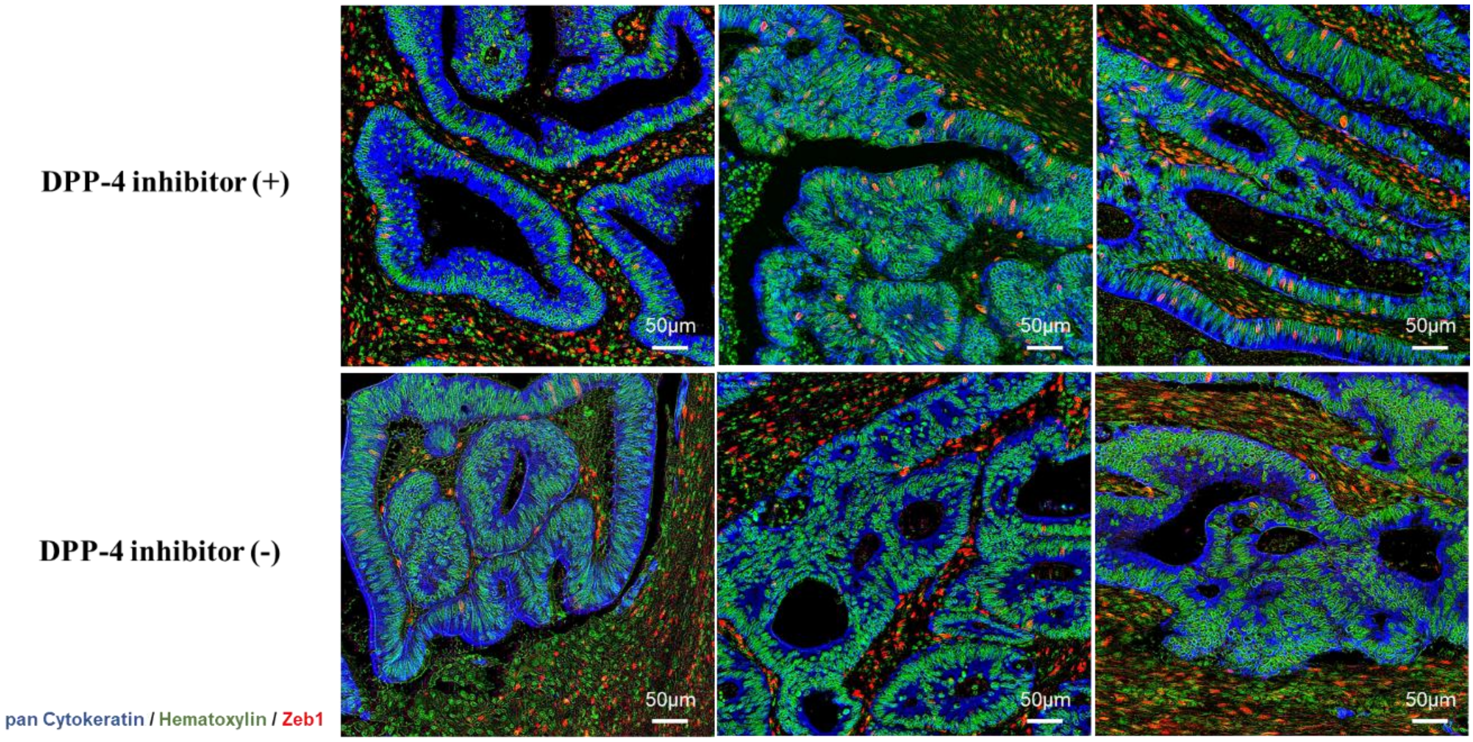

Immunostaining of Cytokeratin (Blue) , Zeb1 (red) and hematoxylin staining (Green) in 3 representative tumors with or without DPP-4i treatment.

Supplementary Figure 5

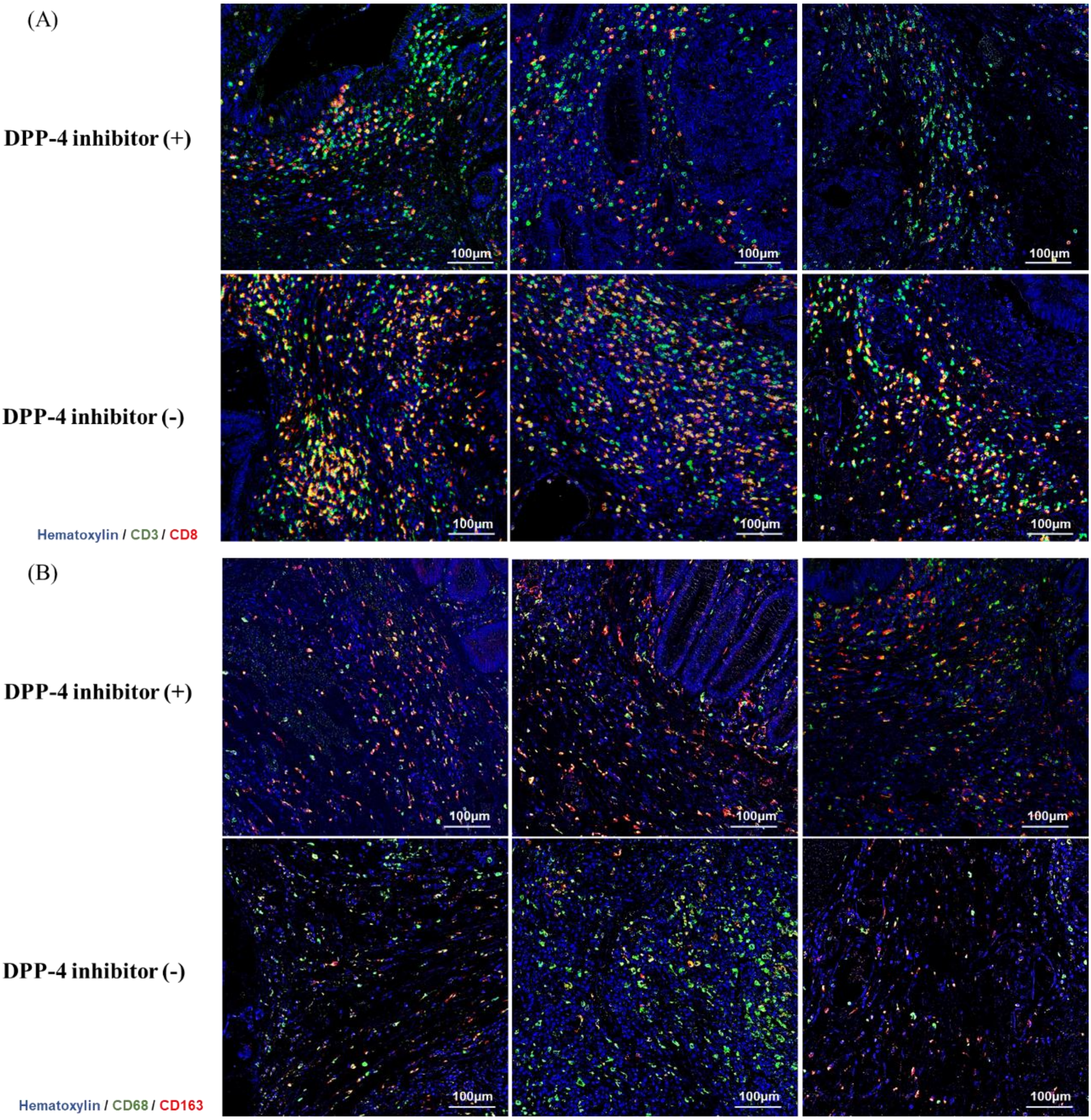

(A) Immunostaining of CD3 (green) and CD8(red) and hematoxylin staining (Blue) in 3 representative tumors with or without DPP-4i treatment.(B) Immunostaining of CD68 (green) and CD163 (red) and hematoxylin staining (Blue) in 3 representative tumors with or without DPP-4i treatment.

Supplementary Figure 6

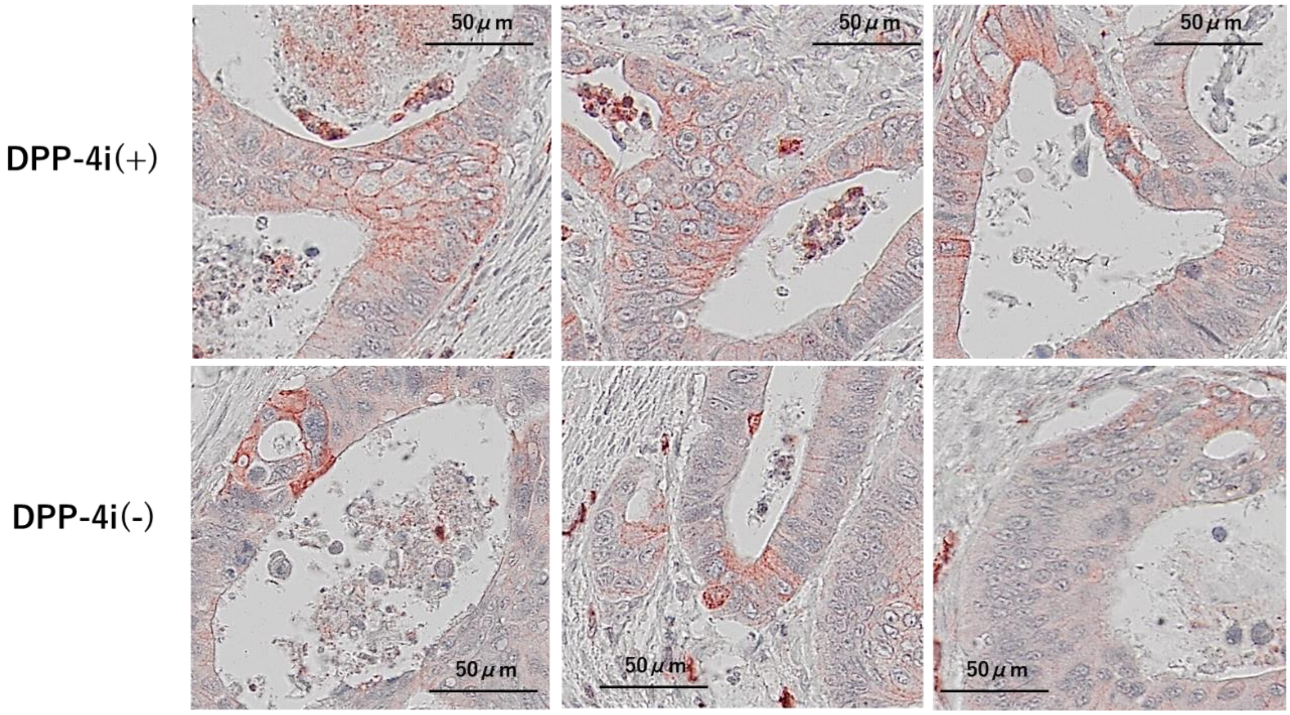

Expression of CXCR4 in CRC tissues in 3 representative tumors with or without DPP-4i treatment. Tissue sections were immunostained with rabbit mAb to CXCR4 (UMB2, Abcam) and visualized with AEC.

Supplementary Figure 7

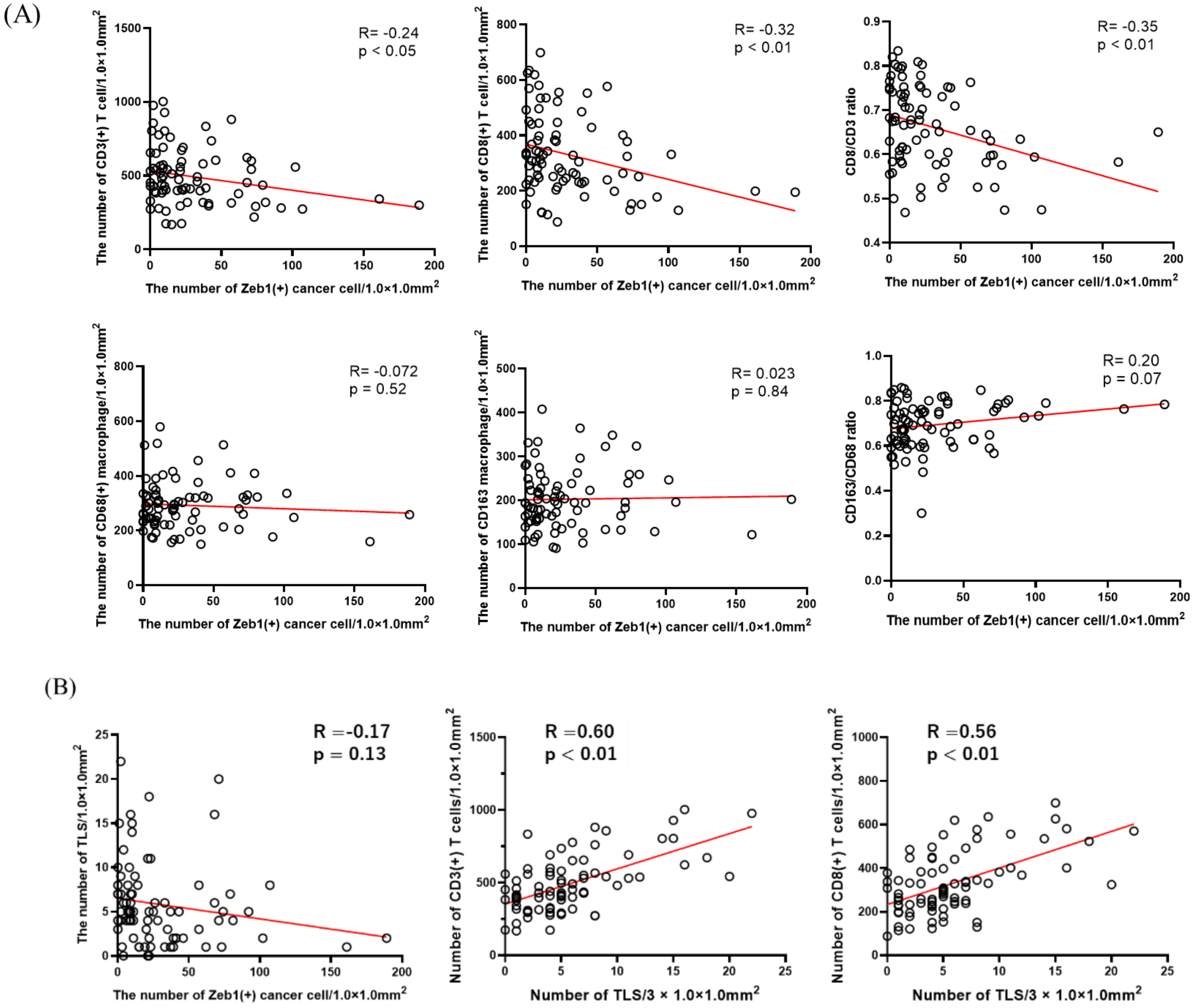

(A) Correlations between Zeb1(+) tumor cells and densities of tumor infiltrating lymphocytes (TILs) and tumor associated macrophages (TAMs) in total of 80 tumors. Correlation coefficients were evaluated with Spearman's rank method.

(B) Correlations between Zeb1(+) tumor cells and tertiary lymphoid structure (TLS) (left) and between TLS and density of tumor infiltrating lymphocytes (TILs) (middle and right) in total of 80 tumors. Correlation coefficients were evaluated with Spearman's rank method.
